# Rational transformative decision-making

**DOI:** 10.1007/s11229-023-04075-9

**Published:** 2023-02-24

**Authors:** Daniel Villiger

**Affiliations:** Institute of Philosophy, Zollikerstrasse 117, 8008 Zürich, Switzerland

**Keywords:** Transformative experiences, Subjective value, Non-subjective value, Desire, Testimony

## Abstract

According to L. A. Paul (2014), transformative experiences pose a challenge for decision theory, as their subjective value is not epistemically accessible. However, several authors propose that the subjective values of options are often irrelevant to their ranking; in many cases, all we need for rational transformative decision-making are the known non-subjective values. This stance is in conflict with Paul’s argument that the subjective value can always swamp the non-subjective value. The approach presented in this paper takes Paul’s argument into account and shows how potential swamping can be controlled given that one desires the transformative outcome: If one knows from previous decisions that desired transformative outcomes are associated with positive subjective value and if, in addition, testimony confirms this association for the current decision situation, one can infer that a desired outcome’s expected subjective value has a positive valence. Accordingly, one can rationally choose the desired transformative option if its non-subjective value is no lower than the overall value of any other option.

## Introduction

Normative decision theory provides guidelines for reaching decision. We take one of the possible options, evaluate its potential outcomes, multiply the value of each outcome with the outcome’s likelihood and add all these products. This gives us the expected value of that option. After we have done this for each of the possible options, we choose the option (or one of the options) with the highest expected value.^1^ This procedure is said to enable rational choice in all decision situations, whether it is what meal you should choose in a restaurant or whether you should become a parent.

In her book *Transformative Experience*, L. A. Paul (2014) rejects the general applicability of this procedure and has thereby triggered immense academic discussions. More precisely, she argues that decision theory is impeded if at least one of the possible outcomes involves a transformative experience. Such an experience has two possible facets. Let us take the outcome of becoming a parent as an example. Becoming a parent is epistemically transformative, since only by becoming a parent do I come to know what being a parent is like and thereby what value it involves. Furthermore, becoming a parent can be personally transformative, changing my very preferences. Because of the transformative character of the option of becoming a parent, we cannot determine its expected value. As a consequence, we are unable to rank our options. The application of decision theory cannot proceed.

However, a back door has been found which (sometimes) still makes rational transformative decision-making possible: An outcome’s transformative character veils its subjective value but not its non-subjective value. The subjective value is an experientially grounded value that includes the assessment of the nature of what it is like to *live* an outcome Paul (2015a, pp. 477–478). In contrast, the non-subjective value includes the outcome’s non-experiential aspects. As proposed by Kauppinen (2015) and Reuter and Messerli (2018), this non-subjective value can be (and often is) sufficient to determine which option’s expected value is highest. Yet, as promising as their approaches using this back door appear at first sight, they are in conflict with a basic feature of transformative experiences as delineated by Paul (2014): the subjective value might always swamp the non-subjective value. So, even an outcome with a highly positive non-subjective value might ultimately have a negative overall value due to an extremely negative subjective value. In turn, this circumstance locks the promising back door.^2^

The present paper offers a new approach that also focuses on a transformative outcome’s non-subjective value but provides controls to ensure that the subjective value does not swamp it. More precisely, rational transformative decision-making where an agent desires the transformative option becomes possible if three conditions are met: (1) the agent knows from prior decisions that desired transformative outcomes are associated with positive subjective value; (2) the agent knows from testimony that people who desired the transformative outcome experienced positive subjective value when the outcome occurred; (3) the transformative option’s non-subjective value is not lower than the overall value of any other alternative. The first two conditions tell the agent that the transformative outcome’s subjective value has a positive valence. This, combined with the third condition, reveals that the expected overall value of the transformative option must be higher than that of the alternatives.

The paper is structured as follows. Section 2 discusses the irrelevance hypothesis endorsed by Kauppinen (2015) and Reuter and Messerli (2018) and shows why it should be rejected. Section 3 then presents a corrective to their approaches. It analyses desires and elaborates on how they can afford a glimpse of a transformative outcome’s subjective value and can thereby make rational transformative decision-making possible.

## The irrelevance hypothesis and its flaw

Reuter and Messerli (2018) and Kauppinen (2015) claim that rational transformative decision-making is often possible despite complete ignorance of the subjective values of transformative outcomes. On their views, information other than the subjective value (i.e. the non-subjective value) can be sufficient to determine the option with the highest expected value. Consequently, the subjective value becomes irrelevant to the decision. This is why these approaches follow what I call *the irrelevance hypothesis*. We will begin with a closer look at Reuter and Messerli’s (2018) non-subjective value approach and then examine Kauppinen’s (2015) story-regarding approach.

The basic idea emphasized by Reuter and Messerli (2018) can already be found in Paul’s (2014) book. Regarding the decision of whether to become a parent, she writes that ‘[i]n the past, non-subjective facts and circumstances played a much larger role in the causal process leading up to parenthood’ (p. 85). This is partly due to the circumstance that contraceptive devices were not available, with the result that people just ended up having children. Yet, even in cases of actively choosing to have children, Paul says that often the motivation was that the person ‘needed an heir, or needed more hands to work the farm, or whatever’ (ibid.). To put it differently, the subjective value of having a child was largely irrelevant, and the decision depended mainly or even exclusively on non-subjective values. However, Paul argues that such a non-subjective approach to transformative decisions appears odd in the context of contemporary affluent Western culture. She contends that dispending with subjective deliberation and subjective values in today’s Western world amounts to rejecting a central principle of that culture’s ordinary way of thinking about choices.

Reuter and Messerli (2018) question the assumed centrality of subjective value to transformative decisions.^3^ To begin with, they use the following example involving a multi-criteria decision model to show how subjective value can become irrelevant to rational decision-making. Mary thinks about whether she wants to have a child and identifies three relevant criteria of varying importance. The most important criterion is (a) whether her partner would like to have a child; 40% of her decision depends on this first criterion. The second-most important criterion is (b) whether she and her partner have the financial means to provide for a child; 35% of her decision depends on this second criterion. Finally, for the third criterion, (c) Mary imagines what it would be like for her to have a child; the remaining 25% of her decision depends on this third criterion. Mary can proceed to give each criterion a value between 0 and 1 for each of the two possible outcomes, namely, becoming a parent and not becoming a parent. On the one hand, she knows the values for criteria (a) and (b) since they are non-subjective. Let us say that her partner wants to become a parent. Therefore, in regard to criterion (a), she assigns a value of 1 to having a child and 0 to not having a child. Additionally, let us assume that their financial means are stable but not great. So, in regard to criterion (b), she assigns the value 0.5 to both having a child and not having a child. On the other hand, the value of criterion (c) is inaccessible to her because becoming a parent is transformative. Nevertheless, if we do the math, we realise that the value for criterion (c) is in fact irrelevant to the final decision. Without considering criterion (c), the having-a-child outcome receives a value of 0.4 * 1 + 0.35 * 0.5 = 0.575, whereas the not-having-a-child outcome receives a value of 0.4 * 0 + 0.35 * 0.5 = 0.175. Consequently, even if the values for criterion (c) turn out to be 0 for having a child and 1 for not having a child, the ranking of the options does not change.^4^ Mary can therefore rationally choose to become a parent even though the subjective value of becoming a parent is not epistemically accessible to her.^5^

In a next step, Reuter and Messerli show that the dynamics of this fictive example about Mary actually apply quite often in real decisions regarding whether to become a parent. First, they asked participants to name the three criteria they would consider most important in making this decision. The five criteria mentioned most often were: consistency with previous goals, subjective value, costs, outcome of discussion with partner and outcome of reading literature. These five criteria plus the criterion of openness to change were then given to another group of participants, who had to weight each criterion according to its importance to the decision of whether to become a parent.^6^ On average, subjective value received only 17% of the overall weight, which implies that it was not more central for participants than other criteria. Since the authors did not collect the values that participants would have assigned to each of the criteria, they used a statistical model to analyse various possible constellations. The analysis showed that ‘[o]n average, the subjective value influences people’s decisions in only 35% of cases. Thus, people have a chance of around 65% to make a rational choice’ (p. 21).

The study by Reuter and Messerli (2018) seems to demonstrate that even in a life-changing transformative decision such as whether to become a parent, the subjective value is often irrelevant to the ranking of options. Consequently, rational decision-making is still possible most of the time despite the presence of a transformative experience. However, the Reuter and Messerli approach works only because it relies on a special characteristic of multi-criteria decision models: The range of values that a criterion can yield is capped and ranges between 0 and 1, with a fulfilled criterion yielding a value of 1 and an unfulfilled criterion a value of 0.^7^ For that reason, we are able both to assign non-subjective values and to know the lowest and highest possible subjective values. But as soon as assigned values no longer simply represent whether a criterion is fulfilled but how much value an outcome provides with respect to that criterion, we are restricted in our attempts to assign both subjective and non-subjective values.

For, on the one hand, if the highest possible utility value is 1 and the lowest is 0, Mary does not know the intervals between these extreme points and an outcome’s non-subjective values. For example, as the financial situation of Mary and her partner is stable but not great, she assigns the seemingly neutral value of 0.5 to both the having-a-child outcome and the not-having-a-child outcome. But 0.5 is only the neutral point between 0 and 1 if the utility space is symmetrical, i.e. the best possible outcome is as positive as the worst possible outcome is negative. Mary does not know whether this is the case (e.g. the neutral point could be 0.4 or 0.6). And even if she knew that the general utility space is tilted towards the negative (cf. Villiger, 2021), she would not know to what extent (e.g. the neutral point could be 0.6 or 0.7). Accordingly, she cannot assign non-subjective values, as the general shape of the utility space is unknown to her, veiling the intervals between extreme points and an outcome’s non-subjective values. On the other hand, if Mary assigns non-subjective values, she does not know the range of possible subjective values (or values in general). For instance, if she defines an outcome that is neither good nor bad as having a value of 0.5 and a good outcome as having a value of 0.8, she does not know the respective values of the worst and best possible outcomes (e.g. are they 0 and 1, respectively, or -2 and 2?). This is why, in both cases, epistemic inaccessibilities prevent Mary from excluding the possibility that an outcome’s subjective value swamps its non-subjective values. As a result, Mary cannot know whether the subjective value is truly irrelevant to the ranking of her options.^8^

Paul (2014) emphasises this point in an example of her own: An agent might choose to become a parent because she desires to have some of her DNA transmitted to a future generation. So, transmitting one’s DNA to a future generation has non-subjective value to that agent. Yet this non-subjective value must be weighed against the subjective value that goes along with becoming a parent. If this subjective value is sufficiently negative, it swamps the positive non-subjective value of leaving a genetic imprint. And since the agent does not know that the subjective value will not be sufficiently negative, she cannot rationally rank her options. More generally, ‘even if other [features of the] outcomes are relevant, the value of the phenomenal outcome [i.e. the subjective value], when it occurs, might be so positive or so negative that none of the values of the other relevant outcomes matter’ (Paul 2015b, p. 17).

As we have seen, Reuter and Messerli circumvent this problem by replacing Paul’s understanding of decision theory, which builds on expected utility theory, with one that builds on multi-criteria decision analysis. But this does not resolve the apparent challenges that transformative experiences pose for an expected utility theoretical understanding of decision theory. For that reason, the non-subjective value approach of Reuter and Messerli (2018) is not fully satisfactory as a demonstration of rational transformative decision-making.

A second objection to Reuter and Messerli’s approach concerns agents’ weighting of an outcome’s subjective and non-subjective values. Chituc et al. (2021) replicated Reuter and Messerli’s study and controlled for whether participants placed low weights on subjective value due to their lack of knowledge about subjective value – a phenomenon called evaluability bias. Their findings support the presence of an evaluability bias, suggesting that agents would place a higher weight on the subjective value if they were able to evaluate it. But these findings trigger an even more fundamental question: Could agents that do not suffer an evaluability bias rationally weigh an outcome’s subjective and non-subjective values? It seems that they could only do so if the weighting of subjective and non-subjective values is outcome independent, as they would then know the weighting from previous outcomes. However, an outcome independent weighting of subjective and non-subjective value seems little convincing. Even if we focus on life choices, why should becoming a parent, changing career or emigrating to a foreign country have the same weighting of subjective and non-subjective values? This constitutes a major problem for Reuter and Messerli’s approach, regardless of the presence of an evaluability bias.^9^

Let us now turn to Kauppinen’s (2015) story-regarding approach. Like Reuter and Messerli (2018), Kauppinen highlights the importance of non-subjective values in transformative decision-making. But unlike Reuter and Messerli, he postulates the normative claim that life choices should be made on a story-regarding basis, rather than on an experience-regarding basis.^10^ We should mainly be concerned about what life choices ‘mean for the successful pursuit of something objectively valuable that builds on our past efforts and experiences, and is consistent with our commitments’ (p. 373). On this view, then, the subjective value of an experience is not of much importance, so its epistemic inaccessibility does not pose a problem in the first place.^11^

Kauppinen provides two arguments for his normative claim. First, he contends that non-subjective values are typically weightier than subjective values. To justify this, he refers back to Mill’s (1863) notion that it is better to be Socrates dissatisfied than a fool satisfied. So, people who have experience of using ‘higher faculties’ prefer a life that incorporates them to one that does not, even if this entails being less happy. Let us assume that this is correct. It is unclear what its implications are in the case of a decision such as whether to have a child. Indeed, being a parent might provide meaning, but there is little evidence that people generally choose to exchange happiness for meaning. If they did, why would there be so many self-help books on happiness, and why would Western society be so hedonistic? It could be objected that meaning is actually a key constituent of happiness (cf. Kauppinen, 2013). But this line of reasoning typically refers to felt meaning (rather than objective meaning), which is part of the epistemically inaccessible subjective value. Kauppinen goes on to propose that starting a family can be seen as the next stage in an evolving relationship. However, I contend that if a couple somehow knew up front that, in their case, starting a family would involve massive unhappiness, they would most likely not opt for it. Deciding to have a baby simply because that is what a couple does when it wants the relationship to grow seems to be poor deliberation. Finally, there is a strong narrative, at least in Western culture, that a woman becomes a ‘real woman’ only when she fulfils her biological destiny and gives birth to a child (Donath, 2015). Women growing up with this cultural narrative are likely to adopt it, at least implicitly. However, it is hard to imagine that the value of going along with this culturally imposed self-narrative outweighs almost any unhappiness that having a child might involve. In general, narratives can misguide, and the idea that the mere value of being consistent with them naturally compensates for the potential unhappiness they produce is not convincing. Therefore, Kauppinen’s (2015) first argument – that non-subjective values are typically weightier than subjective values, regardless of how negative (or positive) these subjective values may be – is not persuasive.

This is where his second argument comes in. He says that, in the long run, ‘the choices we make are unlikely to matter too much to the quality of our experience, at least when the effect is genuinely unpredictable’ (p. 385). Therefore, from a long-term perspective, subjective values can be cancelled out anyway, because they end up roughly the same, regardless of our choices. This is a bold statement for two reasons. First, while Kauppinen acknowledges that there are experiential outcomes to which we do not adjust, he argues that these exceptions do not pose a problem, because we know in advance that they involve low subjective value. For example, an agent knows that the subjective value of caring for a severely disabled child without family and community support is low. Thus, this agent can take this potential outcome and its low subjective value into consideration when she is deciding whether to have a child. Apart from these predictable exceptions, Kauppinen maintains, subjective values generally converge after some time, regardless of the outcome.^12^ However, there are two problems here. (1) Even if an agent knows that a transformative outcome involves low subjective value (e.g. < 0), she does not know how low it is (e.g. is it -1 or -100?) and thus cannot determine the outcome’s approximate subjective value. But this information (and the outcome’s probability) is important for assessing whether it is rational to choose an option despite its risks. Thus, Kauppinen’s argument that non-adjustable experiential outcomes do not pose a problem, since we can anticipate them, is invalid. Merely knowing that an option involves possible outcomes with low subjective value is not per se sufficient to assess whether choosing the option is rational.

(2) There is ample evidence, including longitudinal studies with large sample sizes, that the kind of set point model of happiness Kauppinen is presupposing applies neither to (almost) all people nor to (almost) all life events (e.g. Easterlin, 2003; Fujita & Diener, 2005; Lucas, 2007; Lucas et al., 2003; Luhmann et al., 2012; Luhmann & Intelisano, 2018; Oswald & Winkelmann, 2019). Let us take a closer look at parenthood. Clark and Georgellis (2010) analysed data from the British Household Panel Survey (1996–2006). They found that after a one-year increase for women (but not men) up to and including birth, life satisfaction decreases for both men and women by the time the child is two or more years old and does not rehabilitate (long-run effect of ≥ 5 years). This is more or less consistent with data from the German Socio-Economic Panel survey (1984–2003), as analysed by Clark et al. (2008). They found that ‘[w]hile a recent arrival has a positive effect on women’s life satisfaction but no significant effect on men’s, by the time the child is 2–3 years old, the estimated coefficients turn negative for both sexes and remain so thereafter’ (p. 236). Moreover, the authors also discovered a positive anticipation effect one year before birth for both men and women. So, these two studies suggest that, after a possible initial increase, parents’ levels of happiness and life satisfaction decline after having a child and stay below pre-childbirth levels even years later. This finding is also in line with a meta-analysis conducted by Luhmann et al. (2012).

Of course, not all parents undergo such long-lasting changes in life satisfaction. Analysing the same data as Clark et al. (2008), Galatzer-Levy et al. (2011) sought to identify multiple independent response trajectories within the sample. They found that only 7.2% of parents demonstrated a sustained decline in life satisfaction in response to childbirth, whereas 88.4% of parents did not exhibit long-term effects. Yet it would be hasty to conclude that a dampened subjective value due to parenthood is therefore a rarity. With her book *Regretting Motherhood* (2015), Orna Donath broached a taboo topic and triggered immense echo. For the first time, there was a public discussion about whether choosing to have children is sometimes seen in hindsight as a major mistake. Taking up this issue, a German survey asked 1,228 parents whether they regretted parenthood (Geißler & Laude, 2016). Approximately 20% said yes. Interestingly, the ages of their children at the time of the survey only marginally influenced parents’ answers (children 17 or older formed the oldest age cluster). This may imply that regretting parenthood does not fizzle out over time. But is a low subjective value for parenthood (partially) accountable for parents’ regret? There is no bullet-proof answer to this question, but there are clear hints. On the one hand, the survey reveals that parents who regret parenthood experience substantially less satisfaction from parenting than those who do not regret parenthood. On the other hand, Donath’s (2015) qualitative analysis of regretting motherhood demonstrates that the subjective value of being a parent is closely intertwined with regretting parenthood. Therefore, in view of both longitudinal studies and the debate about regretting parenthood, it seems there is a non-negligible proportion of parents whom parenthood has rendered unhappy (or less happy) for a long time. And this is true even though their respective situations were not as difficult as that of a single parent with a severely disabled child and no family and community support.

Let us examine the second reason why subjective values cannot be cancelled out. Even if, after some months or years, a ‘bad choice’ leads to approximately the same level of subjective value as a ‘good choice,’ those months or years of lower subjective value should still be taken seriously. Otherwise, we could go so far as to say that, in the long run, we are all dead – so why bother about decision-making in the first place? Thus, on the whole, Kauppinen’s (2015) second argument is not convincing, either. We cannot simply omit subjective values from our decision-making process, because (1) outcomes can have unpredictable long-term effects on happiness and (2) possible adaptation might still be preceded by years of unhappiness. Combined with the fact that non-subjective values do not per se outweigh all subjective values, this amounts to grounds for rejecting Kauppinen’s story-regarding approach.

We see that both the Reuter and Messerli (2018) approach and the Kauppinen (2015) approach rely untenably on the irrelevance hypothesis, though for different reasons. This brings us back to Paul’s argument that, when confronted with a transformative decision, we can never know whether the subjective value of an outcome will swamp its non-subjective value. Consequently, a rational route to transformative decision-making that focuses on non-subjective values must still somehow consider the subjective values of the outcomes. If only we were able to catch a glimpse of a transformative outcome’s likely subjective value before experiencing it, we would then be able to assess whether the subjective value might swamp the non-subjective value. The next section discusses the possibility of getting such a glimpse.

## Desires in the context of transformative decisions

When science journalist Bas Kast (2018) asked psychologist Gerd Gigerenzer what his take-home message was after decades of research on decision-making, Gigerenzer told the following story. A friend of his loved two women and did not know which to choose. So, he applied Benjamin Franklin’s decision-making procedure and wrote down all the criteria that mattered to him as well as the respective pros and cons of each candidate in relation to each criterion. For example, he evaluated each one’s beauty, imagined how mindful, kind and interesting each would be, compared to the other, after several years of marriage and so on. After that, he weighted each criterion, multiplied these weights with the ratings from his pros and cons list, added up the values for each option and thereby calculated which woman was associated with the higher expected value. There was only one problem: When he saw the answer, he intuitively knew that it was wrong. Consequently, he ignored the list, followed his heart and was happy with the woman he chose for many years.

This story illustrates common folk wisdom regarding decision-making: We can wrack our brain about an important decision, but in the end, we should trust the answer our body gives us. Following this advice himself, Sigmund Freud put it like this:

'When making a decision of minor importance, I have always found it advantageous to consider all the pros and cons. In vital matters, however, such as the choice of a mate or a profession, the decision should come from the unconscious, from somewhere within ourselves.' (Kast, 2018, p. 80)

What is it exactly that our body tells us? In this paper, I want to focus on desires. Desires involve a particular emotional/mental state of longing for a certain outcome and are strong enough to act upon. At this, they often arise from our unconscious mind without our full knowledge of their underlying sources (Irvine, 2006). It will be useful to differentiate intrinsic desires from instrumental desires (Schroeder, 2015). An intrinsic desire means that we desire an outcome (at least partially) for its own sake. For instance, the desire to see an old friend who lives abroad is most likely an intrinsic desire. In contrast, an instrumental desire implies that an outcome is desired merely as a means to some other end and thus not at all for its own sake. Referring to the previous example, the desire to travel to the country where the old friend lives is most likely an instrumental desire: travelling to that country is desired solely because that outcome is a necessary means to satisfy the intrinsic desire of seeing the old friend.

The precise role of desire in decision-making depends on its theoretical conception (for a review, see Schroeder, 2015). The present paper follows Pollock (2006), who writes, ‘[h]uman beings form desires for various features and then try to achieve them. That is just to say that their desires encode goals. They then engage in means/end reasoning to try to achieve their goals’ (p. 35). Pollock further argues that desires are not the most basic conative state and do not play any direct role in the computation of an outcome’s expected value. Therefore, on the one hand, having a desire for outcome X leads one to act in ways that make outcome X (likely) to occur.^13^ Yet, on the other hand, it does not automatically imply that outcome X involves a high (expected) value. The following example illustrates what this means. A woman with no experience in foreign travel develops a strong desire to engage in it because she believes that she will greatly enjoy it. So, she takes unpaid leave for half a year and books a flight to a foreign country to achieve her goal of travelling abroad and satisfying her desire. But when she actually achieves her goal and the desired outcome occurs, she realises that she does not enjoy it as much as she thought she would. In fact, she does not enjoy it at all and regrets that she did not go on with her life as it was. This example demonstrates that satisfying your desire is not per se rational since desires can be based on unfounded expectations, leading to wrongly anticipated (expected) values.

Let us expand upon desires in the context of transformative decision-making. Since desires encode goals and can thereby point one’s life journey in a specific direction, it is not far-fetched to assume that they might be useful in transformative decisions. While the following deliberations are applicable to all sorts of transformative decisions, we once again use the decision of whether to become a parent as an illustrative example. A large proportion of Western people intrinsically desire to have at least one child. Admittedly, this intrinsic desire is often rather abstract, with people saying they dispositionally desire to have children but are not ready yet. Nevertheless, at some point in their lives, this standing intrinsic desire to have children becomes occurrent. In addition to people who have the intrinsic desire to become a parent, there are also people who desire this merely instrumentally; they do not want to have a child for its own sake but only for instrumental reasons, such as to please their partner or to go along with the norm. Can the consideration of such intrinsic or instrumental desires make it rational to choose to become a parent? In the following pages, I argue that it can, if three requirements are met (at this, the first two requirements concern either intrinsic desires, if the agent intrinsically desires to become a parent, or instrumental desires, if the agent instrumentally desires to become a parent).

The first requirement for rationally deciding to become a parent is the agent’s knowledge, based on past choices, that she can trust her desire. As we know, desiring a certain outcome does not guarantee that the outcome will have high subjective value. Normally, however, people should know from previous decisions how well their prior desires for transformative outcomes have corresponded to the subjective values of these outcomes. This knowledge can then be applied to a new, currently desired transformative outcome. Of course, this does not imply that, in such an application, one can precisely calculate the expected subjective value of a transformative outcome. Rather, one might be able to make a first assessment of its valence, namely, whether the expected subjective value is likely to be positive, neutral or negative.^14^ For example, if you know from past choices that when you satisfied a desire for a transformative outcome it sometimes involved a negative, sometimes a neutral, and sometimes a positive subjective value, your desire seems to be an unreliable predictor of the subjective value’s valence. However, if satisfying desires for transformative outcomes has mostly resulted in outcomes with positive subjective value, yielding a positive subjective value of desired transformative outcomes overall, this could be a pattern that also applies to a newly desired transformative outcome.^15^

We can compare our desires with a signal that triggers and accompanies a decision. Over time, we learn to interpret this signal: Is it only noise, or does it tell us something about the actual value of the desired outcome? If we realise that desires for transformative outcomes accurately predict positive subjective values for these outcomes, we can use this expertise for transformative decisions. This is actually similar to what Paul (2014) calls a higher-order technique. For example, Paul argues that someone who has never eaten durian can nevertheless narrow its range of possible subjective values because she has eaten other fruits before. So, eating other fruits has led to higher-order knowledge of what the experience of eating fruit is like, which an agent can directly apply to eating durian. While this will not reveal the precise phenomenological character of eating durian, it still enables a good enough assessment of its subjective value. The association of desired transformative outcomes with positive subjective values is also a higher-order fact that can be applied to newly desired transformative outcomes.^16^ But unlike the higher-order facts in Paul’s durian case, this higher-order fact does not yet enable a good enough glimpse of a transformative outcome’s subjective value. There is still too much ignorance regarding the ways in which desires associate with subjective value in the specific case of becoming a parent.

The second requirement for rationally deciding to become a parent reduces the ignorance left from the first requirement. It contains that testimony of parents who desired to become a parent indicates that, overall, being a parent provides them positive subjective value. At best, such testimony is well-matched, meaning those who provide testimony were in a similar situation when deciding to have children as the agent is right now. For example, they match in terms of their respective relationships’ stability, financial situations and planned co-parenting models. If available, agents can also consult empirical studies that examine whether desiring to have children affects the subjective value of having children. Relatedly, Su (2012), for instance, studies whether the general finding of parenthood having a negative effect on well-being depends on whether or not pregnancies were intended. Her analysis shows that it does: Intended parents and childless people report similarly positive levels of life satisfaction, which is significantly higher than that of unintended parents (see also Bronte-Tinkew et al., 2009; Claridge et al., 2017). Admittedly, an intention to have a child is not the same as a desire to have a child. Still, the two likely have a tight correlation, suggesting that agents who desire to become parents do not have to expect a drop in life satisfaction after becoming a parent (as the psychological literature normally suggests).

At this point, however, it seems necessary to once again invoke the distinction between intrinsic and instrumental desire. In *Regretting Motherhood*, Donath (2015) depicts the decision-making process of 13 of her 23 subjects. Of these 13 regretful mothers, 11 did not have an intrinsic desire to have children when they became pregnant (or did not deliberate on the decision at all). For example, one of Donath’s subjects, Debra, said, ‘It wasn’t because that’s what I wanted, but it was the price I had to pay for my relationship. […] In fact, ever since I can remember, the subject of family and motherhood didn’t interest me’ (p. 25). Similarly, a subject named Edith reported, ‘I messed up, and had children … because when we got married I had been accepted to medical school and he told me, “Listen, if you’re going to study medicine we’re getting a divorce. I want kids.” And like an idiot, I thought – what do you mean divorce? So what? So I won’t study medicine—what’s the big deal?’ (p. 25). Finally, a subject named Doreen stated, ‘Since the day we got married, he just wouldn’t stop … putting terrible pressure on me, to the point of saying, “Okay, if we’re not going to try and get pregnant, we’re getting divorced.” […] And I said, “Okay, I don’t want a divorce, let’s do it.” But I felt all along that it’s … wrong. … I didn’t feel it was the right thing to do at all. I mean, I didn’t want the second one either’ (pp. 24–25). These quotes and Donath’s qualitative analysis more generally reveal that having children for instrumental reasons can backfire dramatically. Accordingly, these findings question that, overall, parents who instrumentally desired to become a parent yield positive subjective value from being a parent, leaving the second requirement potentially unmet.

The fulfilment of the second requirement suggests that an agent’s learned association of desired transformative outcomes with positive subjective values also applies to the outcome of becoming a parent. This enables one to obtain a glimpse of the transformative outcome’s subjective value that is good enough for assessing its expected valence. In turn, this provides a control to ensure that the subjective value does not swamp the non-subjective value.^17^ Therefore, if the first two requirements are met, we can safely rely on the analysis of an outcome’s non-subjective value, which brings us to the third and last requirement.

The third requirement for rationally deciding to become a parent is that its non-subjective value not be lower than the overall value of any alternative.^18^ The following example illustrates how this, combined with the other two requirements, makes rational transformative decision-making possible. Consider an agent who has the intrinsic desire to become a parent and is in a position to choose between becoming a parent and staying childless. Since the agent knows what it is like to be childless and desires to become a parent, we assume that the overall value of staying childless is negative. In contrast, the epistemically accessible non-subjective value of becoming a parent is positive. If the agent knows (1) from prior decisions that she can trust her intrinsic desire and (2) from testimony that parents who intrinsically desired to have a child experienced positive subjective value, she can expect that the subjective value of becoming a parent will not swamp its positive non-subjective value. As a result, she can rationally choose to become a parent, because its expected overall value is positive, whereas the overall value of staying childless is negative.

What if a desired outcome is personally transformative, as it likely is in the case of becoming a parent? Since this alters the agent’s preferences, we do not know whether the non-subjective value of the desired outcome will turn out differently than expected, which would seem to impede its assessment. However, how people’s preferences change when undergoing a transformative experience need not be random; it may follow some pattern. If it does, an agent can anticipate how her preferences might change and, correspondingly, how the desired outcome’s subjective value would turn out in light of her transformed preferences. By consulting (well-matched) testimony and/or empirical studies, the agent can come to know whether such a pattern of personal transformation exists. For example, regarding having a child, a study conducted by Lönnqvist et al. (2018) finds that women (but not men) tend to become more conservative when entering parenthood, attributing higher value to self-restriction, the preservation of traditional practices and the protection of stability. Therefore, a woman deciding whether to have a child can anticipate that if her preferences change, they are likely to change in that direction. In turn, this information enables her to assess the non-subjective value of having a child, even if it involves a personal transformation.

Finally, let us consider a transformative decision where the third requirement is not met. Suppose an agent has the desire to wreak revenge on someone who wronged her in the past and wants to burn that person’s car. Since she has never done anything like this before, the experience is transformative and thus has an unknown subjective value (unlike the alternative of not burning his car). But she knows from previous decisions that, for her, desired outcomes are associated with positive subjective value. Moreover, for the sake of this argument, let us assume that she also is familiar with testimony indicating that satisfying the desire to wreak revenge involves positive subjective value. Can she therefore rationally choose to burn his car? No, because the non-subjective value of burning his car is most probably lower than the overall value of not burning his car, since burning someone’s car is morally reprehensible. As a consequence, she cannot rationally choose the desired option ‘burning his car’ even though the first two requirements are met.

To summarise, rational transformative decision-making becomes possible in cases where we desire the transformative option, provided that each of the following holds: (1) one knows from prior decisions that one’s desired transformative outcomes are associated with positive subjective value; (2) one knows from testimony that people who desired the transformative outcome experienced positive subjective value when the outcome occurred; (3) the transformative outcome’s non-subjective value is no lower than the overall value of any other alternative. Table 1 illustrates how the three requirements jointly allow for rational transformative decision-making.

**Table 1:** How the three requirements allow a ranking of options despite the presence of a transformative experience. *Note*. $v(X)$ = value of X; $nsv(X)$ = non-subjective value of X; $sv(X)$ = subjective value of X; $TO_{d}$ = desired transformative option; $NA$ = non-transformative alternative.

	Decision Theoretical Implications
Requirements 1 & 2	$sv\left(T{O}_{d}\right)>0$
Requirement 3	$\bigwedge nsv\left(T{O}_{d}\right)\ge v\left(NA\right)$
$\Rightarrow$ Ranking of Options	$\to v\left(T{O}_{d}\right)>v\left(NA\right)$

Five final remarks are in order. First, does the fulfilment of the first two requirements really provide sufficient reason to assume that the desired outcome has a positive expected subjective value? There are several accounts of rational transformative decision-making which argue that consulting testimony by itself enables agents to assess a transformative outcome’s expected subjective value (e.g. Chang, 2015; Dougherty et al., 2015; Pettigrew, 2015, 2016, 2020). If consulting testimony by itself is seen as sufficient to assess a transformative outcome’s expected subjective value, then the fulfilment of the first two requirements should certainly provide sufficient reason to assume that the subjective value does not swamp the non-subjective value. This is because those two requirements are more demanding than merely consulting testimony and only need to enable agents to assess the valence of a transformative outcome’s subjective value (not the value itself, as in the other accounts). Importantly, Paul (2014, 2015a, b) agrees that the consultation of third-personal information can get standard decision theory going again and technically make a ‘rational’ transformative decision possible. However, she has issues with this normative standard of rationality as it comes at the cost of authenticity, which brings us to the next remark.^19^

Second, Paul (2014, 2015a, b) repeatedly argues against consulting testimony as an expedient for assessing a transformative outcome’s subjective value since doing so leads to an inauthentic choice. More precisely, she contends that agents who base their decision on testimony give up their first-personal perspective and become completely dependent on third-personal information, which is untenable. But even though the present paper’s approach also appeals to agents’ reference on testimony, agents do not leave the first-personal perspective on this account. They first-personally (and introspectively) consider what they desire and look at their past decisions to assess whether they can trust their desire. Only in a second step do they consult testimony, in order to learn whether desires were associated with positive subjective value in the case of those who already chose the transformative outcome. So, the third-personal perspective does not replace the first-personal perspective but, rather, complements it, resulting in a combination of introspection (what do I desire), reflection (can I trust my desire) and empirical validation (could others trust their desire in this very decision). This distinction to mere testimony accounts becomes important when looking at Paul’s objections to consulting third-personal information. Paul (2015b, p. 800) criticises the use of testimony because it does not reveal an outcome’s subjective value by one’s own lights but by the lights of the average member of one’s population, which is not the same.^20^ Moreover, testimony cannot distinguish between the future subjective value for the agent who is now making the choice and the future subjective value for the potentially personally transformed agent. The present paper’s account is not affected by either of these objections since it does not replace the agent’s subjective value of an outcome with that of the average member of one’s population. It only checks whether the agent’s learned association of desired transformative outcomes with positive subjective values also applies to the current decision context. Ultimately, Paul does not criticise the use of testimony as a way of anticipating a personally transformative outcome’s non-subjective values under transformed preferences. Consequently, her critique of consulting testimony should not affect the present paper’s account.

Third, it might be objected that the third requirement is hardly fulfillable, making this paper’s account practically unfeasible. For example, regarding the transformative outcome of becoming a parent, it seems to be the potential subjective value of the parent-child attachment relation which makes it attractive. Yet this value is irrelevant to the third requirement. Conversely, regarding the non-transformative outcome of staying childless, it seems to be the non-subjective value of retaining independence, financial stability, etc. that makes it attractive. This value is relevant to the third requirement. With this much in mind, how could the non-subjective value of becoming a parent ever not be lower than the overall value of remaining childless? The answer lies in the subjective value of remaining childless. If an agent desires to become a parent, not becoming a parent turns into a deficiency. Consequently, not satisfying the desire to become a parent comes with low subjective value. This low subjective value can swamp the non-subjective value of remaining childless, making the overall value of remaining childless less or equal to the non-subjective value of becoming a parent.

Fourth, previous decisions involving desired transformative outcomes and testimony might tell us more than simply whether a transformative outcome’s expected subjective value is positive. For example, they might indicate that the expected subjective value of such an outcome is highly positive and thus able to compensate in part for a comparatively low non-subjective value. Accordingly, even if the non-subjective value of a desired outcome is lower than the overall value of an alternative, one might still be able to make a rational decision. Let us suppose that the non-subjective value of a desired outcome is less than the overall value of the alternative by amount X. As long as previous transformative decisions and testimony allow us to infer that the expected subjective value of the desired outcome is greater than X, we can rationally choose it. More generally: if the third requirement is insufficiently fulfilled, it can be compensated for with stronger fulfilments of the first and second requirements (and vice versa). Therefore, there are workable alternative versions of the requirements presented in this paper. The reason I formulated them as I did is that, this way, the first two requirements control potential swamping and thereby disable Paul’s main argument against non-subjective value approaches to rational transformative decision-making.

Fifth, what if both the desired outcome and the alternative(s) are transformative (e.g. an undergraduate career in economics vs. one in philosophy)? In that case, the requirements must be slightly modified so that rational transformative decision-making becomes possible again. The adjusted first requirement is that one knows from prior decisions that a transformative outcome’s desirability positively correlates with its subjective value. The adjusted second requirement is that testimony and/or empirical studies confirm such a correlation regarding the desired transformative outcome. The adjusted third requirement is that the non-subjective value of the desired transformative option is not lower than the non-subjective value of any alternative.^21^

## Conclusion

The approach presented in this paper builds on the importance, emphasised by Kauppinen (2015) and by Reuter and Messerli (2018), of non-subjective values in transformative decision-making. However, in contrast to their attempts to circumvent an outcome’s subjective value, this paper’s approach imposes direct controls to ensure that the outcome’s subjective value will not swamp its non-subjective value. More precisely, desires can afford a glimpse of a subjective value’s valence if prior decisions have shown that desired transformative outcomes are associated with positive subjective values and testimony confirms this association in the current decision situation. This allows for rational transformative decision-making whenever the non-subjective value of a transformative option is no lower than the overall value of any of the other options.

## Data Availability

Not applicable.

## Footnotes

1 In the case of ordinary reasoners, this decision-making process is approximative (Paul, 2015c).

2 The differentiation between subjective and non-subjective values (sometimes also called phenomenal and non-phenomenal values or experiential and non-experiential values) has been introduced by Paul (2014) and constitutes a fundamental and well-accepted aspect in the debate on transformative experiences (cf. Barnes, 2015; Bykvist & Stefánsson, 2017; Chang, 2015; Kauppinen, 2015; Reuter & Messerli, 2018; Sharadin, 2015; Talbott, 2016; Villiger, 2021). While this differentiation and its implications can of course be criticised, the goal of the present paper is to make a contribution within the debate on transformative experiences. This is why I adopt Paul’s differentiation between subjective and non-subjective values, including her general idea that subjective values can swamp non-subjective values (also consider that there are arguments for the differentiation between subjective and non-subjective values and for the possibility of swamping: see e.g. Paul, 2014, pp. 42–44, 2015a, pp. 477–479, 532–533, 2015c, p. 165; Reuter & Messerli, 2018, pp. 333–334; Sharadin, 2015, pp. 443–444).

3 Reuter and Messerli (2018) operationalise the subjective value by using the criterion ‘I imagine what it will feel like to have the experiences and emotions when being a parent’ (p. 18). Therefore, they roughly equate subjective value with the value of subjective feel. In this regard, it has to be mentioned that Paul (2015a, p. 513) rejects the reduction of subjective value to the value of subjective feel. Still, it remains unclear what Paul would consider an appropriate operationalisation of subjective value since her own definition of subjective value as the value of what it is like to *live* an outcome is rather vague.

4 Regarding criterion (c), if the value of not having a child is 1, we attain an overall value of 0.425 for that outcome. This is in any case lower than the overall value of the having-a-child outcome, which comes to at least 0.575.

5 Reuter and Messerli acknowledge that this might cease to be the case if becoming a parent is also personally transformative. This is because a change in preferences might affect the weighting of the three criteria.

6 Reuter and Messerli discuss whether the what-it-is-like criterion referring to an outcome’s subjective value can actually be separated from the other criteria. For example, it could be argued that when assessing a choice’s financial consequences, which are said to involve non-subjective value, agents ultimately imagine how it feels to live in good or bad financial conditions. The authors conclude that various criteria have little to no bearing on the subjective value of becoming a parent, including costs, outcome of discussion with partner, outcome of reading literature and openness to change. Still, it might not always be easy to separate the what-it-is-like criterion that refers to an outcome’s subjective value from other criteria that refer to its non-subjective value.

7 The value range in a multi-criteria decision model need not be 0–1. However, assigning different lowest and highest possible values does not affect the argument.

8 Let us resume Reuter and Messerli’s example of Mary deliberating on whether to have a child, where the (a) asking-partner criterion has a weight of 40%, the (b) cost criterion has a weight of 35% and the (c) what-it-is-like criterion has a weight of 25%. Regarding the having-a-child outcome, Mary assigns a value of 1 to (a) and a value of 0.5 to (b), leading to a combined value of 0.575. Regarding the not-having-a-child outcome, Mary assigns a value of 0 to (a) and a value of 0.5 to (b), leading to a combined value of 0.175. Given that 0 and 1 truly constitute the lowest and highest possible values, respectively, the outcomes’ subjective value is irrelevant, and Mary can reach a rational decision (best possible value of not-having-a-child outcome = 0.425; worst possible value of having-a-child outcome = 0.575). But what if the lowest possible subjective value were not 0 but some number below − 0.6 (while keeping 1 as the highest possible subjective value)? In that case, the outcomes’ subjective values would not be irrelevant (best possible value of not-having-a-child outcome = 0.425; worst possible value of having-a-child outcome < 0.425). Since Mary cannot know that the lowest possible outcome is not below − 0.6, she does not know whether the outcomes’ subjective values are irrelevant; thus, she cannot reach a rational decision.

9 There seems to be a rational way out here. Agents can inform their weighting of subjective and non-subjective values by consulting empirical studies and/or (well-matched) testimony from people who have already experienced the relevant transformative outcome.

10 This circumvents both problems of Reuter and Messerli’s non-subjective value approach.

11 Kauppinen (2015) argues that the subjective value is broadly hedonistic and thus more or less equal to hedonic value. Paul (2015a, pp. 513–517) rejects this definition of subjective value as being too restrictive.

12 Kauppinen (2015) mentions impact bias as evidence for his argument. Impact bias is the tendency to overestimate how positive or negative one’s affective reactions to a certain outcome will be (Wilson & Gilbert, 2003). However, this does not automatically imply that affective reactions converge after some time regardless of the outcome. So, the existence of impact bias does not establish Kauppinen’s claim.

13 Of course, we can also resist a desire. So, a desire does not automatically lead to desire-satisfying behavior.

14 We can set the following anchor to define positive, neutral and negative subjective values: the subjective value of zero is associated with experiences that, overall, are neither positive nor negative and thus can be described as being neutral. Consequently, any overall good experience has greater than zero subjective value, and any overall bad experience has less than zero subjective value (cf. Villiger, 2021).

15 In such a situation, desired transformative outcomes would be associated with positive subjective value. For example, an agent has so far experienced ten desired transformative outcomes and their subjective values were 6, 5, 4, …, -2, -3. In that case, more often than not, desired transformative outcomes provided positive subjective value (six out of ten times), yielding a positive subjective value of such outcomes overall (on average 1.5). Consequently, there is an association of desired transformative outcomes with positive subjective value.

16 We could say that this is an indirectly applicable higher-order fact, because the experiences that the higher-order fact embraces resemble each other not in their phenomenology but in their transformative character. Moreover, we could divide desired transformative outcomes into different subcategories such as ‘desired transformative outcomes in the context of career’, ‘desired transformative outcomes in the context of family’, etc. These differentiated desired transformative outcomes might then also be associated with different subjective values.

17 To provide such control, how high must the credence for the proposition ‘overall, those who desired to have children experienced positive subjective value when having children’ be to sufficiently reduce the ignorance left from the first requirement? While there is no clear answer to this question (and no similar discussion in other accounts of rational transformative decision-making, cf. Barnes, 2015; Chang, 2015; Dougherty et al., 2015; Pettigrew, 2015, 2016, 2020; Sharadin, 2015; Villiger, 2021), I make the following suggestion. The required credence should depend on how strong the agent’s association between desired transformative outcomes and positive subjective value is: the weaker this association, the higher the required credence must be. The cumulated positive subjective value of desired transformative outcomes divided by the modulus of the cumulated negative subjective value of desired transformative outcomes provides a proxy for how strong the association between desired transformative outcomes and positive subjective value is (let us call this the ‘ATS’ and its inverse the ‘iATS’). To sufficiently reduce the ignorance left from the first requirement, the required credence for the proposition ‘overall, those who desired to have children experienced positive subjective value when having children’ must be greater than $\frac{{1 + iATS}}{2}$. Following this formula, the required credence must be close to 1 if the association is very weak and just above 0.5 if the association is very strong. To resume the example of footnote 15, the iATS would be $\frac{{3 + 2 + 1}}{{6 + 5 + 4 + 3 + 2 + 1}} = \frac{2}{7}$, leading to a required credence for the proposition ‘overall, those who desired to have children experienced positive subjective value when having children’ of greater than $\frac{{1 + 2/7}}{2} \approx 0.64$.

18 For the moment, we assume that all alternatives are non-transformative and that we therefore know their overall value. At the end of the paper, we will see how rational transformative decision-making is also possible with transformative alternatives. Additionally, when I say that we know the value of an outcome/alternative, I mean that we either know the actual value or can calculate the expected value.

19 In one passage, Paul (2015b, p. 795) questions whether transformative decision-making by means of testimony can be ‘rational’ in the first place as it replaces the decision problem with a different one: the agent does no longer need to consider the possible phenomenal character of the transformative experience but only its potential value. However, there are several reasons why this objection can be neglected. First, Paul’s own revelatory account to rational transformative decision-making does not consider the possible phenomenal character of the transformative experience either. Second, she writes that ‘reflective replacement of one’s introspective assessment with an expert’s assessment’ might be possible (p. 811). Thus, considering the possible phenomenal character of the transformative experience does not seem to be a strict necessity for rational decision-making. Third, multiple times, Paul emphasises the conflict between rational and authentic choice. For example, in her reply to Pettigrew’s account which grounds on testimony, she writes that ‘he has drawn out just how deep and fundamental the divide between deciding rationally and deciding authentically may be’ (p. 794). In her book (Paul, 2014), she mentions that we face a Sartrean dilemma when reaching a transformative decision: ‘either choose authentically, or choose rationally’ (p. 112). Finally, in another reply, Paul (2015a, pp. 493, 497, 498, 499) repeatedly refers to the fundamental/central tension between rationality and authenticity in (some) transformative choices. Therefore, Paul seems to agree that you can make a transformative choice rationally, however, it comes at the cost of authenticity and for Paul (2014) dropping authenticity ‘is untenable to the extreme’ (p. 112).

20 Besides, at the moment, third-personal information about how much subjective value an agent is expected to get when choosing X or Y is still very imprecise in big life choices.

21 What if there are both transformative and non-transformative alternatives to the desired transformative option? In that case, both the original and the adjusted first and second requirement must be fulfilled. In addition, the original third requirement must be met in relation to all non-transformative alternatives, and the adjusted third requirement must be met in relation to all transformative alternatives.
